# Perspectives on resting-state functional magnetic resonance imaging research in vascular dementia

**DOI:** 10.3389/fnagi.2025.1547965

**Published:** 2025-07-04

**Authors:** Jinhuan Yue, Peng Wang, Xiang-fa Guo, Zeyi Wei, Kaiting He, Nuo Li, Xiaoling Li, Qinhong Zhang, Xiaoqing Zhou

**Affiliations:** ^1^Shenzhen Frontiers in Chinese Medicine Research Co., Ltd., Shenzhen, China; ^2^Vitality University, Hayward, CA United States; ^3^Division of Oncology, First Affiliated Hospital of Heilongjiang University of Chinese Medicine, Harbin, China; ^4^Department of Medical Imaging, Second Affiliated Hospital of Heilongjiang University of Chinese Medicine, Harbin, China; ^5^Institute of Acupuncture and Moxibustion, Shandong University of Traditional Chinese Medicine, Jinan, China; ^6^Department of Acupuncture, Beijing University of Chinese Medicine Shenzhen Hospital (Longgang), Shenzhen, China; ^7^Division of CT and MRI, First Affiliated Hospital of Heilongjiang University of Chinese Medicine, Harbin, China; ^8^Heilongjiang University of Chinese Medicine, Harbin, China

**Keywords:** vascular dementia, resting-state functional magnetic resonance imaging, amplitude of low-frequency fluctuations, regional homogeneity, functional connectivity

## Abstract

Vascular dementia (VD) is a severe cognitive impairment syndrome resulting from various cerebrovascular diseases and is one of the leading causes of senile dementia. In recent years, its incidence has been steadily increasing. Given the lack of specific treatments for VD, early detection, diagnosis, and intervention are critically important. This review examines the existing literature on resting-state functional magnetic resonance imaging (rs-fMRI) in the context of VD, with a focus on key metrics such as amplitude of low-frequency fluctuations (ALFF), regional homogeneity (ReHo), and functional connectivity (FC). By analyzing changes in brain functional activity in VD patients as observed through rs-fMRI, this study aims to provide essential imaging insights that could support and enhance clinical treatment strategies.

## 1 Introduction

Dementia is a syndrome characterized by a variety of symptoms, including memory loss, impaired judgment and reasoning, as well as changes in emotion, behavior, and communication (Smith and Buckwalter, 2005). Vascular dementia (VD) is the second most common form of dementia after Alzheimer’s disease (AD), accounting for approximately 15% of all dementia cases (O’Brien and Thomas, 2015). VD encompasses various types of vascular lesions and can be classified into several subtypes based on the nature of the vascular pathology and its clinical manifestations (O’Brien and Thomas, 2017). Compared to AD, VD is more commonly associated with prominent deficits in executive function and attention, while memory impairment is less pronounced (Román et al., 2002). Despite the significant need, there are currently no specific treatments available for VD (McShane et al., 2019; Craig and Birks, 2005; Chapman et al., 2004). A deeper understanding of the neurochemical changes related to cognitive impairment and imaging alterations in patients with cerebrovascular disease is essential for developing targeted and effective treatments for VD (Kirvell et al., 2010; Jagust et al., 2008).

Resting-state functional magnetic resonance imaging (rs-fMRI) is a technique that involves placing subjects in a relaxed state with their eyes closed, minimizing any structured thought processes, while using blood oxygen level-dependent signals to measure changes in brain function (Ten Kate et al., 2018). This method is used to detect spontaneous brain activity during rest, enabling researchers to explore brain function and identify regions of activation in response to specific neural activities (Wang et al., 2019a; Wang et al., 2019b; Wei et al., 2023). The primary rs-fMRI techniques include amplitude of low-frequency fluctuations (ALFF), regional homogeneity (ReHo), and functional connectivity (FC) (Hao et al., 2022; Li et al., 2021). An essential consideration in rs-fMRI research is the signal-to-noise ratio (SNR), which plays a pivotal role in determining the reliability and interpretability of imaging results (Liu, 2016). Variability in SNR can arise from factors such as scanner performance, data acquisition settings, and preprocessing methods, potentially impacting the precision of key metrics like ALFF, ReHo, and FC. Efforts to optimize SNR are crucial for enhancing the detection of resting-state brain activity and ensuring that analyses yield reproducible and meaningful insights (DeDora et al., 2016).

To contextualize the role of rs-fMRI in vascular dementia research, several critical distinctions must be emphasized. VD represents a heterogeneous diagnostic category encompassing cognitive impairment attributable to diverse cerebrovascular pathologies, whereas subcortical ischemic vascular dementia (SIVD) constitutes a specific VD subtype characterized by small vessel disease markers including white matter hyperintensities and lacunar infarcts. This nosological precision is essential, as SIVD demonstrates distinct neuropsychological profiles (prominent executive dysfunction with relative memory preservation) and rs-fMRI signatures compared to other VD subtypes or AD (O’Brien and Thomas, 2017; Román et al., 2002). The three primary rs-fMRI metrics discussed herein - ALFF, ReHo, and FC - provide complementary insights into brain network dysfunction. ALFF quantifies the magnitude of spontaneous neuronal activity within individual regions, with alterations in specific frequency bands (e.g., slow-5 [0.01–0.027 Hz]) reflecting either vascular dysregulation or compensatory mechanisms (Zou et al., 2008; Li et al., 2014). ReHo measures the temporal synchronization of neighboring voxels, serving as a sensitive marker of local network integrity that correlates with domain-specific cognitive deficits in VD (Tu et al., 2020). FC maps the coordinated activity between distributed brain regions, revealing characteristic patterns of network disintegration that differentiate VD from other dementias (Buckner et al., 2009; Zhang et al., 2013). These metrics derive physiological validity from multimodal studies demonstrating convergence between rs-fMRI patterns and histopathological changes (Jagust et al., 2008), though researchers must remain cognizant of the inferential limitations inherent in correlational neuroimaging approaches.

## 2 FC

Functional connectivity (FC) is a critical rs-fMRI metric that measures the exchange of functional information between anatomically distinct brain regions, providing insights into the coordination of neural activity across the brain (Buckner et al., 2009; van den Heuvel and Hulshoff Pol, 2010). FC has been extensively studied in neurodegenerative conditions, including VD and AD, to understand the alterations in brain network interactions associated with these disorders.

Functional connectivity analyses in vascular dementia reveal distinct patterns of network disintegration that reflect underlying cerebrovascular pathophysiology. Unlike AD’s predominant default mode network disruption, VD typically exhibits more severe disconnection in frontal executive networks and subcortical-cortical loops (Zhang et al., 2013; Fu et al., 2019). The emerging technique of voxel-mirrored homotopic connectivity (VMHC) has demonstrated particular promise in differentiating VD from AD, with VD patients showing characteristic reductions in interhemispheric synchronization of orbital and straight gyri - regions especially vulnerable to hypoperfusion (Cheung et al., 2021). Frequency-specific FC analyses further enhance diagnostic precision, as slow-5 band connectivity appears uniquely sensitive to microvascular pathology (Li et al., 2014). These network-level abnormalities correlate meaningfully with clinical features; for example, disrupted connectivity within the salience network associates with apathy in SIVD, while dorsal attention network disturbances correlate with impaired vigilance (Altunkaya et al., 2022). Such findings underscore FC’s potential as both a diagnostic tool and a means of elucidating structure-function relationships in vascular cognitive impairment.

Zhang et al. (2013) investigated FC patterns across different frequency bands in patients with VD using whole-brain FC mapping. Their study revealed that FC varies significantly across three frequency bands (slow-5, slow-4, and full frequency band), each with different capacities to distinguish between brain states. Notably, the slow-5 frequency band showed the highest ability to differentiate VD brains from healthy controls, suggesting that this band is particularly sensitive to the functional disruptions associated with VD. These findings highlight the potential of multivariate pattern analysis methods to detect FC abnormalities in VD across varying frequency bands, which could enhance the understanding of VD pathogenesis.

Altunkaya et al. (2022) explored FC differences between patients with SIVD and AD, focusing on apathy-related changes. The study identified distinct alterations in FC both within and between resting-state networks in SIVD and AD (Altunkaya et al., 2022). Shared FC changes were observed within the dorsal attention network, while differing patterns were identified within the salience network. Using a region-specific approach, the study highlighted the right inferior frontal gyrus, left middle frontal gyrus, and left anterior insula as key hubs linked to the “initiating” deficits that contribute to apathy.

The “disconnection theory” referenced here pertains to the disruption of FC between critical brain regions and networks, which compromises the brain’s ability to integrate and process information effectively (Dai et al., 2019). This concept has been widely applied in neurodegenerative diseases to explain cognitive and behavioral deficits arising from network-level disintegration. In the context of SIVD and AD, disconnections often involve weakened FC within and between key networks such as the default mode network (DMN), dorsal attention network, and salience network (Dai et al., 2019). These disruptions can manifest as distinct clinical symptoms; for example, apathy in SIVD may be linked to impaired FC in frontal and subcortical regions, while AD-related deficits often involve the medial temporal lobe and DMN (Dai et al., 2019). This framework highlights the importance of exploring FC disruptions as underlying mechanisms for disorder-specific cognitive and behavioral impairments. Further studies are needed to validate these patterns and explore their diagnostic and therapeutic implications. These findings extend the disconnection theory by incorporating the impact of FC interactions across multiple RSNs on the development of apathy in neurodegenerative diseases.

Cheung et al. (2021) employed VMHC to assess interhemispheric FC as a diagnostic tool for distinguishing VD from other AD-related neurodegenerative conditions. The study identified specific VMHC patterns when comparing patients with VD, AD, and mild cognitive impairment (MCI) to healthy controls. In VD patients, significant reductions in VMHC were found in the orbital and straight gyri, while increased VMHC was observed in regions associated with the DMN, executive control network (ECN), and salience network. Conversely, AD patients exhibited decreased interhemispheric FC across all DMN, ECN, and SN regions, whereas MCI patients showed reduced VMHC in the SN and increased VMHC in the DMN and ECN. Fu et al. (2019) examined static functional network connectivity (sFNC) and dynamic functional network connectivity (dFNC) across 54 intrinsic connectivity networks in 19 patients with AD, 19 patients with SIVD, and 38 age-matched healthy controls. The results revealed that in both patient groups, the sFNC between the visual regions and the cerebellum was increased, while the sFNC between the cognitive control regions and the cerebellum was decreased (Fu et al., 2019). SIVD specifically showed a reduction in the sFNC within the sensorimotor domain, whereas AD was associated with alterations in the sFNC between the DMN and the cerebellum regions (Fu et al., 2019). Additionally, SIVD was characterized by a higher frequency and longer duration of weak connectivity states in dFNC, and a lower frequency and shorter duration of strong connectivity states (Fu et al., 2019). The findings highlight both shared and distinct functional connectivity changes in AD and SIVD from static and dynamic perspectives, and suggest that dFNC may serve as a more significant biomarker for dementia.

**FIGURE 1:**
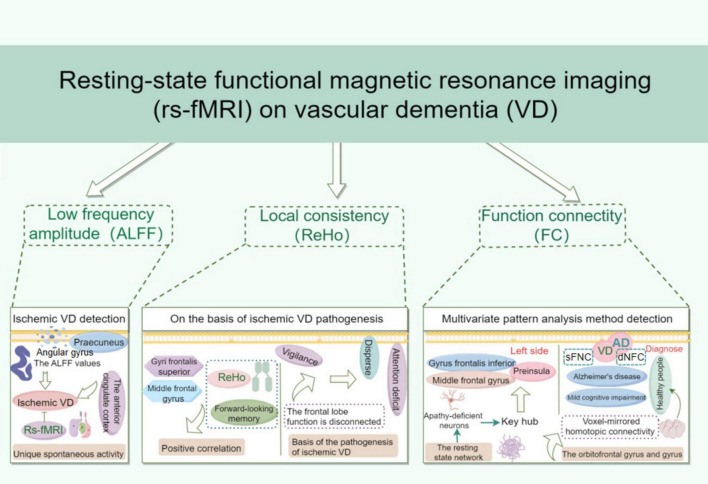
Resting-state functional magnetic resonance imaging on vascular dementia.

Incorporating VMHC values from relevant brain regions, the receiver operating characteristic analysis demonstrated that VMHC could accurately differentiate healthy controls from VD, AD, and MCI with accuracies of 87%, 92%, and 83%, respectively (Figure 1). These findings suggest that VMHC serves as a reliable diagnostic tool for distinguishing between VD, AD, and MCI by examining VMHC patterns and values in specific brain regions (Figure 1). This approach not only aids in differential diagnosis but also provides a deeper understanding of the distinct interhemispheric connectivity alterations associated with these neurodegenerative conditions.

## 3 ALFF

The amplitude of low-frequency fluctuations (ALFF) is a novel metric used to assess the magnitude of spontaneous brain activity during rs-fMRI. ALFF is calculated by taking the square root of the power spectrum within a predefined low-frequency range, providing a quantitative measure of the intensity of brain activity in specific frequency bands (Zou et al., 2008; Zang et al., 2007).

The interpretation of ALFF findings in vascular cognitive impairment requires careful consideration of frequency band specificity. The preferential use of slow-5 (0.01–0.027 Hz) and slow-4 (0.027–0.073 Hz) bandwidths is grounded in their distinct neurobiological correlates: slow-5 oscillations demonstrate particular sensitivity to microvascular pathology, as evidenced by their strong association with white matter lesion burden in SIVD (Li et al., 2014), whereas slow-4 activity may reflect compensatory reorganization in relatively preserved brain regions. This frequency-dependent stratification enables more precise localization of vascular pathophysiology, as exemplified by the selective correlation between slow-5 ALFF in the right angular gyrus and activities of daily living scores in SIVD patients (Li et al., 2014). Such findings underscore the importance of bandwidth-specific analysis protocols when investigating cerebrovascular contributions to cognitive decline.

Research by Zuo et al. (2010) and Han et al. (2011) explored the spatial distribution of ALFF across two frequency bands: slow-5 (0.01–0.027 Hz) and slow-4 (0.027–0.073 Hz). Their studies revealed considerable variability in ALFF values across different brain regions, highlighting significant differences between these two frequency bands. These findings underscore the importance of considering frequency-specific characteristics when analyzing resting-state brain activity.

Further investigations (Wee et al., 2012; Salvador et al., 2008; Malinen et al., 2010; Baria et al., 2011; Baliki et al., 2011) have emphasized the role of different frequency bands in shaping the overall characteristics of whole-brain functional networks and associated brain states. These studies suggest that frequency-specific alterations in ALFF can provide deeper insights into the functional organization and dynamics of brain networks.

Liu et al. (2014) examined alterations in ALFF across the entire brain in patients with SIVD. Compared to healthy controls, both SIVD patients and controls exhibited significantly elevated standardized ALFF values in regions including the bilateral anterior cingulate cortex (ACC), posterior cingulate cortex, medial prefrontal cortex, inferior parietal lobule, occipital lobe, and adjacent precuneus. However, when directly compared to the control group, SIVD patients demonstrated lower ALFF values specifically in the bilateral precuneus, along with higher ALFF values in the bilateral ACC, left insula, and hippocampus. Li et al. (2014) analyzed the ALFF in two distinct frequency bands in 30 patients with SIVD: slow-5 (0.01–0.027 Hz) and slow-4 (0.027–0.073 Hz). In the slow-5 band, SIVD patients exhibited significantly higher ALFF in the bilateral anterior cingulate cortex, right putamen, and right supplementary motor area compared to the control group, while the right precuneus and right angular gyrus showed significantly lower ALFF in the patient group (Li et al., 2014). Moreover, ALFF in the right angular gyrus was found to be closely correlated with the Activities of Daily Living scores (Li et al., 2014). In the slow-4 band, SIVD patients demonstrated increased ALFF in the bilateral anterior cingulate cortex, right putamen, and left fusiform gyrus, but no significant correlation was observed with cognitive performance scores (Li et al., 2014). These findings suggest that SIVD patients exhibit widespread abnormal intrinsic neural oscillations, which appear to be dependent on specific frequency bands. Specifically, ALFF in the right angular gyrus within the slow-5 band appears to be more specific to SIVD, suggesting its potential as a valuable diagnostic marker for the disease.

These findings suggest that SIVD is characterized by distinct spontaneous abnormalities in resting-state brain activity, as reflected in ALFF measurements. Notably, alterations in ALFF values in the precuneus, ACC, insula, and hippocampus may serve as potential biomarkers for detecting SIVD (Figure 1). This underscores the clinical utility of ALFF in understanding the pathophysiology of SIVD and potentially aiding in its diagnosis.

## 4 REHO

Regional homogeneity (ReHo) is a rs-fMRI metric that evaluates local FC by assessing the similarity of the time series of a given voxel with those of its neighboring voxels. ReHo has been demonstrated to be sensitive to neurodegenerative processes, including AD and VD (Tu et al., 2020; Liu et al., 2008).

ReHo’s utility as a biomarker for vascular cognitive impairment stems from its sensitivity to localized network disruptions characteristic of small vessel disease. In SIVD, reduced ReHo in the dorsolateral prefrontal cortex correlates strongly with executive dysfunction (Tu et al., 2020), likely reflecting the disruption of frontal-subcortical circuits by ischemic white matter lesions. Conversely, elevated ReHo in posterior cortical regions may indicate maladaptive functional reorganization or loss of inhibitory control. These patterns demonstrate regional specificity when compared to AD, which typically shows more pronounced ReHo alterations in medial temporal and default mode network regions (Liu et al., 2008). However, interpretation requires caution given potential confounders including head motion artifacts and the partial volume effects common in elderly populations with cerebrovascular disease (Liu, 2016). The correlation between frontal ReHo abnormalities and prospective memory deficits in SIVD (Zhuang et al., 2021) provides particularly compelling evidence for the metric’s clinical relevance, though future studies incorporating longitudinal designs are needed to establish causal relationships.

Prospective memory (PM) is the cognitive ability to remember and execute planned intentions at an appropriate future moment (Román-Caballero and Mioni, 2023). It is typically divided into two subtypes: time-based PM, which requires initiating an action at a predetermined time, and event-based PM, which involves responding to specific external cues (Román-Caballero and Mioni, 2023). Impairments in PM are frequently observed in neurodegenerative disorders, where disruptions in functional connectivity and localized brain activity often play a role. Given its sensitivity to neurofunctional changes, ReHo is a valuable tool for investigating the neural underpinnings of PM deficits in conditions like vascular dementia.

Zhuang et al. (2021) investigated PM performance in patients with early-stage SIVD, comparing their performance with that of AD patients and cognitively normal elderly individuals. The findings by Zhuang et al. (2021) highlight significant correlations between PM performance and ReHo values in specific brain regions in SIVD patients. These results provide important insights into the functional connectivity disruptions underlying memory impairments. However, the lack of citations to similar studies raises questions about the reproducibility and broader relevance of these findings. For instance, Tu et al. (2020) also reported disrupted functional connectivity in neurodegenerative conditions, lending partial support to these observations. To fully establish the significance of these findings, future research should explore similar analyses across diverse cohorts and neurodegenerative disorders, ensuring that the results are validated and contextualized within the broader literature. The study found that the SIVD group exhibited significantly lower PM hit rates on both time-based and non-focal event-based tasks compared to the control group. Interestingly, only the SIVD group, and not the AD group, showed significantly worse performance than the control group in the very early stages of the disease.

Further correlation analysis revealed that in SIVD patients, performance on non-focal event-based PM tasks was positively correlated with ReHo values in the bilateral superior frontal gyrus and middle frontal gyrus. However, time-based PM performance did not show a significant correlation with ReHo in any dorsomedial frontoparietal regions of interest. These findings suggest that SIVD patients’ vulnerability in non-focal event-based PM is linked to disruptions in local FC within the bilateral superior and middle frontal gyri.

Extensive research underscores the significant overlap between episodic memory (EM) and PM, particularly in AD. They are closely interdependent cognitive systems that share common neural substrates, including the medial temporal lobe, prefrontal cortex, and the DMN. These regions are particularly vulnerable to degeneration in AD, leading to impairments in both EM and PM. Disruptions in the DMN, a hallmark feature of AD, have been consistently linked to deficits in both memory systems, suggesting a strong interconnection between them. Research has increasingly demonstrated the overlap between EM and PM in AD. For instance, Kinsella et al. (2007) showed that deficits in EM in early AD are closely related to impairments in PM. This finding underscores the idea that these two memory systems do not function independently, but are rather intertwined, with deficits in one potentially exacerbating deficits in the other. Supporting this view, Román-Caballero and Mioni (2023) found that both time-based and event-based PM tasks are significantly impaired in AD, further suggesting that PM deficits in AD are not isolated but closely linked to other cognitive declines, particularly those affecting EM. The interdependence of these memory systems is further corroborated by studies by Graa and Ergis (2021) and Eusop-Roussel and Ergis (2008) who found that PM deficits in AD may be indicative of broader dysfunctions in the episodic memory system. These findings support the notion that PM impairments may not be purely isolated, but rather an extension of generalized memory system dysfunction in AD.

Beyond AD, this overlap between EM and PM has also been observed in other neurodegenerative diseases. For example, Zhuang et al. (2021) found similar deficits in both EM and PM in patients with SIVD. This indicates that the interdependence between these two memory systems is not unique to AD but is a broader feature of neurodegenerative disorders, reinforcing the importance of considering the overlap between EM and PM in clinical and research settings. Understanding how these memory systems interact could provide valuable insights into the cognitive dysfunctions observed in AD and related conditions.

Given the complexity of these interactions, further research is required to explore how the relationship between EM and PM varies across different neurodegenerative diseases. It is essential to identify the underlying mechanisms driving these deficits, particularly how functional connectivity disruptions contribute to memory impairments in these diseases.

Tu et al. (2020) examined attention distribution and FC in the frontal regions of patients with SIVD and AD using voxel-based ReHo analysis of rs-fMRI data. The study identified significant clusters of ReHo in frontal regions and found that although SIVD and AD patients demonstrated comparable overall cognitive abilities, SIVD patients performed worse on tasks requiring divided attention and vigilance/sustained attention compared to AD patients.

Compared to the normal control group, SIVD patients exhibited reduced ReHo in the right middle frontal gyrus and the left anterior cingulate gyrus, while AD patients showed increased ReHo in the right orbital frontal region. These results indicate that the patterns of FC disruptions differ between SIVD and AD, reflecting distinct underlying pathophysiological mechanisms.

The study by Tu et al. concluded that the poorer attention performance observed in SIVD patients, compared to AD patients, is associated with disrupted FC in frontal regions, specifically in the right middle frontal gyrus and left anterior cingulate gyrus (Tu et al., 2020) (Figure 1). This frontal lobe disconnection may underlie the mechanisms leading to deficits in divided attention and impairments in vigilance/sustained attention, contributing to the significant variability observed within the SIVD patient group (Figure 1). These findings underscore the importance of ReHo as a sensitive measure of local FC disruptions, which can aid in differentiating between dementia subtypes and understanding their distinct cognitive deficits.

## 5 Limitations

This study, together with the existing body of research on VD, faces several noteworthy limitations that constrain both the interpretability of findings and the advancement of clinical translation. First, many studies are limited by small sample sizes, reducing statistical power and limiting the generalizability of observed results. Larger, multicenter studies with diverse patient cohorts are necessary to validate and extend current conclusions. Second, the predominance of single-modality imaging approaches, particularly rs-fMRI alone, restricts comprehensive insight into the multifaceted pathophysiology of VD. Integrating multimodal techniques—such as combining rs-fMRI with structural MRI, positron emission tomography, or diffusion tensor imaging—would allow for a more holistic understanding of both vascular contributions and neurodegenerative progression. Third, the field’s heavy reliance on cross-sectional designs limits our ability to track disease progression and causal relationships. Given the dynamic and evolving nature of cerebrovascular disease, longitudinal studies are essential to differentiate primary vascular effects from downstream neurodegenerative changes and to assess treatment impact over time. Fourth, despite growing interest in non-pharmacological interventions, the potential therapeutic role of psychotherapeutic and behavioral strategies in VD remains insufficiently explored. Such interventions may offer meaningful benefits in emotional regulation, cognitive resilience, and quality of life, and merit systematic evaluation through controlled clinical studies. Fifth, rs-fMRI-based research faces several conceptual and technical challenges. A significant concern is the prevalent use of reverse inference—linking cognitive dysfunction to specific network alterations—without pathological confirmation or interventional validation. Moreover, the frequent conflation of VD subtypes (e.g., subcortical ischemic vs. multi-infarct) and the co-occurrence of AD’s pathology complicate the interpretation of rs-fMRI metrics such as FC, ReHo, and ALFF, particularly when band-specific effects are considered. Additionally, inconsistencies in rs-fMRI acquisition parameters, such as variable frequency definitions in ALFF analysis, hinder the comparability and reproducibility of findings across studies. Compounding this issue is the often suboptimal SNR in aging or clinically impaired populations, which limits sensitivity to subtle neural changes. Addressing these challenges will require the development of standardized acquisition protocols, methodological harmonization, and technological advances such as real-time noise correction and adaptive filtering. Finally, this mini-review itself is subject to inherent limitations, including potential selection bias in the literature reviewed and a narrowed focus on rs-fMRI-derived metrics, possibly overlooking relevant data from other imaging modalities. Furthermore, the reliance on a limited number of key studies, such as Zhuang et al. (2021), underscores the need for further confirmatory research involving larger, independent samples and head-to-head comparisons across cohorts. Future research should prioritize longitudinal, multimodal, and pathologically validated approaches to more accurately characterize network-level disruptions in VD and guide evidence-based therapeutic strategies.

## 6 Summary

Resting-state functional magnetic resonance imaging (Rs-fMRI) has emerged as a critical tool for exploring human brain function due to its non-invasive nature, ease of use, and ability to provide objective insights with high participant compliance. In the context of VD, rs-fMRI studies have utilized analytical metrics such as amplitude of ALFF, ReHo, and FC to identify significant alterations in brain activity.

Key findings include changes in brain regions such as the precuneus, anterior cingulate cortex, insula, and hippocampus. These studies have also highlighted frequency-specific variations in ALFF, emphasizing the importance of analyzing distinct frequency bands. ReHo values in the superior and middle frontal gyri have been correlated with PM performance, while abnormalities in frontal lobe regions have been linked to attentional deficits. Additionally, FC disruptions have been observed in major brain networks, including the dorsal attention network, salience network, and DMN, reflecting the intensity and specificity of brain activity in VD.

These insights not only enhance our understanding of the neurobiological mechanisms underlying VD but also provide valuable imaging evidence for diagnosis and therapeutic interventions. As the global population ages, the role of advanced neuroimaging techniques in early diagnosis and screening of VD will become increasingly significant. Future research into the neurobiological underpinnings of VD will likely drive further innovations in clinical applications, ultimately improving patient outcomes.
